# Biological Activities of Reactive Oxygen and Nitrogen Species: Oxidative Stress *versus* Signal Transduction

**DOI:** 10.3390/biom5020472

**Published:** 2015-04-15

**Authors:** Adelheid Weidinger, Andrey V. Kozlov

**Affiliations:** Ludwig Boltzmann Institute for Experimental and Clinical Traumatology, AUVA Research Center, Donaueschingenstraße 13, 1200 Vienna, Austria; E-Mail: adelheid.weidinger@trauma.lbg.ac.at

**Keywords:** superoxide radical, hydrogen peroxide, hydroxyl radical, nitric oxide, mitochondria

## Abstract

In the past, reactive oxygen and nitrogen species (RONS) were shown to cause oxidative damage to biomolecules, contributing to the development of a variety of diseases. However, recent evidence has suggested that intracellular RONS are an important component of intracellular signaling cascades. The aim of this review was to consolidate old and new ideas on the chemical, physiological and pathological role of RONS for a better understanding of their properties and specific activities. Critical consideration of the literature reveals that deleterious effects do not appear if only one primary species (superoxide radical, nitric oxide) is present in a biological system, even at high concentrations. The prerequisite of deleterious effects is the formation of highly reactive secondary species (hydroxyl radical, peroxynitrite), emerging exclusively upon reaction with another primary species or a transition metal. The secondary species are toxic, not well controlled, causing irreversible damage to all classes of biomolecules. In contrast, primary RONS are well controlled (superoxide dismutase, catalase), and their reactions with biomolecules are reversible, making them ideal for physiological/pathophysiological intracellular signaling. We assume that whether RONS have a signal transducing or damaging effect is primarily defined by their quality, being primary or secondary RONS, and only secondly by their quantity.

## 1. Introduction

Reactive oxygen and nitrogen species (RONS) include two classes of chemically-reactive molecules containing oxygen (reactive oxygen species, ROS) and nitrogen (reactive nitrogen species, RNS). Both classes are referred to as RONS. The majority of RONS carries unpaired electrons and is called free radicals. In mammalians, a major function of specialized enzymes, such as NADPH-oxidase, myeloperoxidase and nitric oxide synthase (NOS), is the generation of RONS. The controlled generation of RONS in the extracellular space by these enzymes was developed evolutionarily as part of the innate immune system to kill bacteria. However, an overwhelming release of RONS may also induce deleterious effects, causing damage to host biological structures. Another group of enzymes release RONS intracellularly as a byproduct of metabolic processes. For instance, superoxide (O_2_^•^^−^) is released as a byproduct of mitochondrial respiration and monooxygenase activity of cytochrome p450. Intracellular RONS, as well as excessive release of extracellular RONS were thought to induce deleterious effects, causing oxidative damage to different kinds of biomolecules. These processes are referred to as “oxidative stress”.

Oxidative stress is believed to significantly contribute to the development of a number of diseases, particularly age-related diseases. However, more and more evidence suggest that intracellular generation of RONS is an important component of intracellular signaling cascades regulating several physiological functions, such as regulation of vascular tone, insulin synthesis, activation of hypoxia-inducible factor (HIF), cell proliferation, differentiation and migration. It took over 50 years for a clear understanding of the chemical basis of free radical/RONS biology to emerge. In the following 50 years, studies on the biological effects of free radicals with biological targets were undertaken. The aim of this review is to put together old and recent ideas on the chemical and pathophysiological role of RONS for a better understanding of their properties and specific activities. This review is predominantly based on selected reviews, elaborating on different aspects of RONS activity and thought to be a guide through a large body of literature existing on this topic.

## 2. Chemical Basics

The current knowledge on RONS biology is based on studies of free radical reactions conducted more than 100 years ago. Free radicals are defined as molecules having an unpaired electron. Their physical properties are similar to those of free electrons, giving a signal at *g* = 2.0023 in the electron paramagnetic resonance spectrum [1]. The chemical mechanisms underlying the formation and toxicity of free radicals were proposed by the British chemist Henry J. H. Fenton in 1894 and later developed by the Austrian chemist Joseph Weiss and the German chemist and Nobel Prize winner Fritz Haber in 1934. Henry J. H. Fenton showed that the formation of toxic hydroxyl radicals (^•^OH) from hydrogen peroxide (H_2_O_2_) is catalyzed by iron ions, called the “Fenton reaction” ([2], reviewed in [3]). He pointed out that iron ions are necessary to form toxic ^•^OH radicals. Joseph Weiss and Fritz Haber discovered that O_2_^•−^ can be converted into H_2_O_2_ and further to ^•^OH, called the Haber–Weiss reaction ([4], reviewed in [5,6]). This reaction shows that one free radical can give rise to another secondary radical. Already in those days, the transformation of one ROS (O_2_^•−^) to another (^•^OH) was associated with the presence of iron ions as a catalyst (reviewed in [7]). Later, other transition metals, such as copper ions, were shown to generate toxic RONS.

Another important step in understanding the biological function of RONS was the discovery of free radical chain reactions. This was done in 1935 by the Russian chemist and Nobel Prize winner Nikolai Semenov. He described four types of free radical reactions, namely initiation, propagation, branching and termination [8]. The same reactions occur in biological membranes upon pathological conditions and are termed lipid peroxidation, the major mechanism of oxidative damage to biological membranes. Importantly, the branching chain reaction of lipid peroxidation, the cleavage of lipid peroxides, is catalyzed by ferrous ions similar to the Fenton reaction, which is a cleavage of H_2_O_2_ to ^•^OH by ferrous ions. The branching chain reaction between lipid peroxides and iron ions accelerates lipid peroxidation [9], again suggesting that iron ions are the prerequisite for the toxic effects of lipid peroxidation.

In the 1950s, researchers started to associate free radical chemistry with biomedical questions. It has been suggested that most of the damaging effects of oxygen in living systems are due to the formation of free radicals (reviewed in [10]). This assumption promoted the application of the knowledge of free radical chemistry to biological systems.

## 3. Oxidative Stress

However, in 1968, a major breakthrough in the field of free radical biology was done by Irvin Fridovich who discovered superoxide dismutase (SOD), a specific enzyme catalyzing the transition of O_2_^•−^ into H_2_O_2_ ([11,12], reviewed in [13]). A few years later, Chance and coauthors reported that mitochondria are the key generator of O_2_^•−^ in cells ([14], reviewed in [15]). These two findings are crucial, as they show that free radicals, on the one hand, are produced in biological systems, and on the other hand, there is an enzymatic mechanism regulating their concentration. This clearly suggests that free radicals occur in biological systems and probably have a specific function. Since then, numerous studies have been performed to understand the biological function of free radicals.

Until the mid-1970s, the literature almost exclusively refers to free radicals. Later, it became evident that not only free radicals, but also non-radical products, such as H_2_O_2_ or hypochlorous acid (HOCl), which are also powerful oxidizing agents, participate in free radical reactions (reviewed in [16]). To take into account both the radical and the non-radical species, the more general term “reactive oxygen species” (ROS) was introduced. Later, nitric oxide (NO^•^) and peroxynitrite (ONOO^−^) were also shown to interact with ROS, and all of these species were termed RONS. Primarily, the toxic properties of RONS were of interest. It was shown that an excessive generation of RONS damaged almost all classes of biomolecules, such as lipids [17], proteins [18] and DNA (reviewed in [19,20]). In the 1970s and 1980s, the term “oxidative stress” was used for these deleterious processes. Later, “oxidative stress” was defined by the German biochemist Helmut Sies as an imbalance between oxidants and antioxidants in favor of the oxidants, potentially leading to damage ([21], reviewed in [22]). Evolutionarily, the induction of oxidative stress was possibly developed as an important part of the innate immune system as a defense mechanism against bacteria [23]. However, it was also shown that RONS, produced by the immune system, can also damage host cells [24].

Careful observation of oxidative damage reactions of biomolecules shows that primary RONS, such as O_2_^•−^, H_2_O_2_ or NO^•^, in most cases reversibly react with the target molecules. NO^•^, for instance, reversibly binds to heme proteins, whereas O_2_^•−^ reacts with proteins, changing their redox state without damage to their structure. For instance, the reaction between O_2_^•−^ and cytochrome c results in the reduction of heme, which is used to detect O_2_^•−^ [25]. The damage is predominantly associated with secondary RONS, such as ^•^OH, ONOO^−^ and HOCl [26,27,28]. All of these toxic species are formed if more than one reactive species is present. Two major reactions leading to the formation of toxic RONS are: (i) the Fenton reaction between ferrous ions and H_2_O_2_ yielding ^•^OH; and (ii) the reaction of O_2_^•−^ with NO^•^ yielding ONOO^−^. Furthermore, the formation of HOCl, formed in an enzymatic reaction from H_2_O_2_ and Cl^−^, is associated with damage to host tissues [28]. In addition, iron ions can directly react with organic peroxides, inducing lipid peroxidation. Moreover, the presence of iron and copper ions induces double-strand breaks of DNA, a DNA damage that is difficult to repair (reviewed in [29]). Some anticancer drugs are based on mechanisms causing double-strand breaks of DNA catalyzed by transition metals [30]. Oxidative damage to proteins is often associated with the reaction between amino acids and ONOO^−^, resulting in the formation of nitrated amino acids, such as nitrotyrosine. ONOO^−^ is a secondary RONS formed in the reaction between NO^•^ and O_2_^•−^. ONOO^−^ and NO^•^ may have quite opposite biological effects. For instance, NO^•^ has an inhibitory effect on lipid peroxidation, while ONOO^−^ activates this process (reviewed in [31]). Another important regulatory mechanism based on the interaction of NO^•^ and O_2_^•−^ is driven by decreased NO^•^ levels, rather than by increased ONOO^−^ levels (reviewed in [32,33]). The interaction between NO^•^ and O_2_^•−^ might also result in vasoconstriction by inactivation of prostacyclin synthase (reviewed in [34]).

The data described above suggest that primary ROS (O_2_^•−^, NO^•^, H_2_O_2_) only have a weak damaging potential, whereas secondary RONS are more toxic. Primary species are well controlled by SOD, catalase and NO synthases, while secondary species are less controllable, since there is no specific enzymatic system controlling their levels.

Interestingly, O_2_^•−^-controlling systems are different inside the cells and in extracellular fluids. SOD, the intracellular enzyme for removing O_2_^•−^, and the extracellular SOD (ecSOD) produce potentially dangerous H_2_O_2_. H_2_O_2_ itself is relatively inactive, but can lead to the formation of toxic ^•^OH. In contrast, ceruloplasmin in the blood inactivates O_2_^•−^, yielding H_2_O [35]. However, extracellular SOD, which mainly occurs in tissue ([36,37]; reviewed in [38,39]), can also be found in plasma under specific pathological conditions [40] and contribute to the elimination of O_2_^•−^. The fact that SOD, not ceruloplasmin, occurs in cells indirectly suggests that H_2_O_2_ may have a physiological function inside cells, but not in extracellular fluids. The data gathered in the last few decades suggest that primary RONS formed in mitochondria (O_2_^•−^ and H_2_O_2_) and NO^•^ are associated with intracellular signaling cascades. Since NO-mediated signaling pathways have already been extensively reviewed ([41,42,43,44,45,46,47]), in this review, we focus on non-NO-mediated signaling pathways.

## 4. Signaling

There is a solid body of literature supporting the essential role of mitochondrial ROS in intracellular signaling. The data on the involvement of mitochondrial ROS in intracellular signaling pathways related to inflammation have been summarized in recent reviews [48,49,50]. In the last few years, the role of ROS in positive and negative regulation of insulin signaling has also been intensively studied and reviewed [51]. Furthermore, the role of mitochondrial ROS in activation of HIF has been intensively studied and debated (reviewed in [52,53]). In addition, the role of ROS has been demonstrated for NF-κB-dependent gene transcription and a number of other signaling cascades (reviewed in [54]). Notably, most of the publications on oxidative stress referred to specific types of RONS involved in oxidative damage, whereas data on signaling are predominantly addressed to ROS and RONS in general. This led to a large knowledge gap on the mechanisms of intracellular signaling concerning RONS, since is not clear whether all or only specific RONS contribute to these signaling pathways. In the following section, we will focus on studies where specific types of RONS contributing to intracellular signaling cascades were determined.

## 5. Superoxide Radical

A number of reports suggest O_2_^•−^ as part of intracellular signaling cascades. This species is predominantly produced by mitochondrial complexes I and III (reviewed in [55]). Evidence of the involvement of mitochondrial O_2_^•−^ in intracellular signaling cascades can be shown by:

1. Alteration of mitochondrial function, particularly of complexes I and III, by pharmacological or genetic modulation, which has an effect on signaling pathways.
2. The correlation of a certain O_2_^•−^ level with an effective signaling cascade.
3. Application of mitochondria-targeted antioxidants (mtAOX) or radical scavengers has an effect on signaling pathways.
4. Genetic manipulation of mitochondrial SOD and cytoplasmic SOD decreases the efficiency of specific signaling cascades.

Mitochondrial O_2_^•−^, the primary mitochondrial ROS, was often associated with the regulation of inflammatory pathways, such as activation of the inflammasome, regulation of inflammatory cytokines synthesis and mechanisms of innate immunity. The involvement of mitochondrial O_2_^•−^ in the activation of the inflammasome was suggested by Zhou *et al.* [56]. The authors showed that specific inhibition of mitochondrial complexes I and III, the major sources of ROS in mitochondria, significantly diminished the activation of the “nucleotide-binding domain, leucine-rich family and pyrin domain containing 3” (NLRP3) inflammasome, suggesting that mitochondrial O_2_^•−^ is involved in this signaling cascade. Another important feature of inflammation is the release of cytokines. Bulua *et al.* [57] showed that LPS-stimulated IL-6 production could be reduced by treatment with MitoQ, a mitochondria-targeted radical scavenger. This effect was coincident with increased levels of mitochondrial O_2_^•−^, suggesting a key role of O_2_^•−^ in this signaling pathway. Weidinger *et al.* demonstrated that mitochondria-targeted antioxidants reduce the expression of IL-6 and iNOS in a model of systemic inflammatory response induced by LPS [58]. In leukocytes, Kröller-Schön [59] showed that elevated mtROS formation activated NADPH-oxidase at the posttranslational level. Inhibition of the mitochondrial permeability transition pore, which is supposed to facilitate the transport of O_2_^•−^ from mitochondria to the cytoplasm, prevented activation of NADPH-oxidase. In contrast, the deficiency of mitochondrial SOD intensified the stimulation of NADPH-oxidase, suggesting that this process is mediated by O_2_^•−^, rather than by H_2_O_2_. Applying specific mitochondrial inhibitors and direct detection of mtROS, Dikalov *et al.* suggested that mtROS, presumably O_2_^•−^, are able to activate NADPH-oxidase via activation of protein kinase C (PKC) [49]. These data suggest that mitochondrial O_2_^•−^ orchestrate cellular ROS production upon inflammation. The same group has shown that stimulation of endothelial cells with angiotensin II elevates mitochondrial O_2_^•−^ levels and simultaneously the activity of NADPH-oxidase in this non-immune cell type [60]. However, treatment with mitoTEMPO, a mitochondria-targeted antioxidant, or overexpression of mitochondrial SOD captured O_2_^•−^ and decreased the activation of vascular NADPH oxidases [60]. NADPH oxidases, in turn, may regulate important cellular processes, such as cell migration [61], differentiation [62] and proliferation [63] (reviewed in [64]). These data again suggest that O_2_^•−^ rather than H_2_O_2_ is involved in this signaling cascade. However, in the past, it was believed that O_2_^•−^ does not participate in signaling, as it cannot exit mitochondria due to its polarity. Consequently, H_2_O_2_ formed from O_2_^•−^ was considered a necessary intermediate of O_2_^•−^-mediated actions. H_2_O_2_ is a nonpolar molecule and can easily diffuse through the membranes. Recently, however, the situation has shifted. O_2_^•−^ has been shown to leave mitochondria via the mitochondrial permeability transition pore [65] and anion channels [66]. We also showed that O_2_^•−^ can be released from mitochondria by using O_2_^•−^-sensitive spin probes and electron spin resonance spectroscopy [67]. These data strongly support the postulation that O_2_^•−^ can directly contribute to intracellular signal transduction pathways.

Nevertheless, other groups propose H_2_O_2_ being the RONS-based messenger in intracellular signaling cascades, as well. In contrast to O_2_^•−^, H_2_O_2_ is a neutral molecule and relatively inactive. Thus, H_2_O_2_ is able to cover relatively large distances, up to several cell diameters, before it reacts with its target or is catabolized [68]. Therefore, it is considered as a suitable ROS-dependent signaling component.

## 6. Hydrogen Peroxide

In the literature, the impact of H_2_O_2_ on intracellular signaling is supported by the following evidence:

1. Exogenous H_2_O_2_ has a direct effect on signaling cascades.
2. The H_2_O_2_ level correlates with the effectiveness of intracellular signal transduction.
3. Genetic manipulation of catalase has an effect on signaling.
4. Upregulation of MnSOD and/or Cu/ZnSOD activates signaling.

Treatment with H_2_O_2_ increased the proliferation of endothelial cells in a study of Chen *et al.*, suggesting that H_2_O_2_ directly interferes with pathways regulating proliferation [69]. Wang *et al.* [70] showed that overexpression of the mitochondria-targeted catalase construct suppressed vascular endothelial growth factor (VEGF)-induced cell migration, suggesting the involvement of H_2_O_2_ in the regulation of cell migration. Schmidt *et al.* [71] demonstrated that overexpression of catalase in cell lines caused a deficiency in the activation of NF-κB in response to tumor necrosis factor (TNF), while the catalase inhibitor, aminotriazole, restored the induction of NF-κB. Overexpression of Cu/Zn-dependent SOD elevated NF-κB activation. These data suggest H_2_O_2_ rather than O_2_^•−^ as the mediator of NF-kB pathway activation. Brunelle *et al.* [72] demonstrated that overexpressing glutathione peroxidase or catalase, but not SOD, stabilized HIF-1 in cells, suggesting that H_2_O_2_ acts as signaling molecule in the process of HIF-1 regulation. West *et al.* [73] showed that overexpressing catalase in mitochondria results in impaired bacterial killing by leukocytes, suggesting the predominant role of H_2_O_2_. Hoarau *et al.* [74] demonstrated that H_2_O_2_ plays an essential role in the development of β-cells, as it activates the ERK1/2 pathway. Other studies on H_2_O_2_-mediated signaling are summarized in several reviews [75,76,77].

## 7. Secondary RONS

The majority of papers on RONS-mediated intracellular signaling suggest either O_2_^•−^ or H_2_O_2_ as the major signaling molecule. Only a few studies suggest that signaling molecules may be derivatives of H_2_O_2_. Garlid *et al.* [78] studied ROS-mediated opening of mitochondrial ATP-sensitive potassium channels and suggested an unknown derivative of H_2_O_2_ as a contributor to this pathway. Others suggested that HOCl may participate in extracellular, but not in intracellular signaling, for instance by interaction with TGF-ß1 [79]. A few more publications can be found on peroxynitrite-mediated signaling (reviewed in [80,81]). It has been assumed that peroxynitrite has an impact on pathways, which, under physiological conditions, are regulated by tyrosine phosphorylation and dephosphorylation. ONOO^−^ causes tyrosine nitration, which blocks the respective signaling cascades. Tyrosine nitration seems to have a significant impact on a number of pathways, such as MAP kinase, STAT3, ERK and PKC-mediated pathways (reviewed in [80,81]). The fact that ONOO^−^ irreversibly binds to proteins has a pathological impact on cellular function, rather than contributing to physiological intracellular signaling. This suggests that the biological impact of primary and secondary RONS is different. Primary RONS are predominantly associated with signaling, whereas secondary RONS are associated with oxidative stress. Primary RONS are regulated by SOD, catalase and peroxidases and have a specific physiological function for the regulation of intracellular signaling. The secondary RONS were evolutionarily developed for extracellular actions, predominantly as part of the innate immune system for killing bacteria. The intracellular release of such secondary RONS leads to deleterious consequences, as these are catalytically highly active, without a reliable control system for intracellular levels. We assume that, in evolution, the primary species were developed for intracellular physiological signaling and the secondary species for extracellular actions, such as killing of bacteria. However, at the same time, these species are able to cause damage to the cell.

## 8. Conclusions

**Figure 1:**
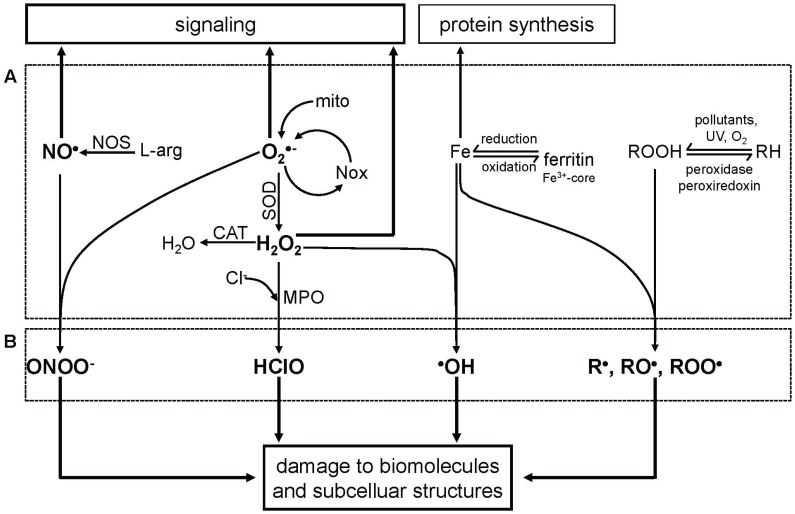
Scheme illustrating physiological and pathophysiological reactions of different reactive species. A, primary reactive species (NO^•^, O_2_^•−^, Fe, ROOH) and the products of the interaction of two identical reactive species (dismutation of O_2_ to H_2_O_2_) and transition metals (reactive oxygen, nitrogen and metal species = RONMS). B, secondary products of reactions between two different RONMS. Primary products predominantly contribute to physiological processes (e.g., signaling, protein synthesis); secondary products exert deleterious effects on diverse cell functions. Abbreviations: NO, nitric oxide; O_2_^•−^, superoxide; Fe, iron; ROOH, lipid peroxide; H_2_O_2_ hydrogen peroxide; RH, non-oxidized lipid; R^•^, RO^•^, ROO^•^, lipid radicals; NOS, nitric oxide synthase; l-arg, l-arginine; ONOO^−^, peroxynitrite; NOX, NADPH oxidase; mito, mitochondria; SOD, superoxide dismutase; CAT, catalase; H_2_O, water; Cl^−^, chloride ion; MPO, myeloperoxidase; HClO, hypochlorous acid; ^•^OH, hydroxyl radical; UV, ultraviolet radiation.

We assume that whether RONS have a beneficial or deleterious effect is primarily defined by their quality, being primary or secondary RONS, and only secondly by their quantity (Figure 1). Therefore, we think that the common statement that at low concentrations ROS regulate physiological processes and at high concentrations are deleterious is not completely correct. Critical consideration of the existing literature shows that deleterious effects, termed as oxidative stress, do not appear if only one primary species is present in a biological system, even at high concentrations. To develop deleterious effects, a primary species reacts with another or a transition metal, yielding highly reactive secondary species, such as ONOO^−^ or ^•^OH. The secondary RONS are catalytically very active, not tightly controlled and consequently may not act as signal transducers. In contrast, primary RONS are well controlled; their reactions with targets are reversible; and they do not damage target molecules. This makes them ideal for intracellular signaling processes. Unfortunately, the majority of papers on signaling refers to RONS without specifying their types. In this review, we highlight an approach allowing one to distinguish the contribution of different RONS. This can be used to define the origin of RONS contributing to intracellular signaling cascades in future studies.
